# Autocatalytic Sets and Assembly Theory: A Toy Model Perspective

**DOI:** 10.3390/e26090808

**Published:** 2024-09-22

**Authors:** Sebastian Raubitzek, Alexander Schatten, Philip König, Edina Marica, Sebastian Eresheim, Kevin Mallinger

**Affiliations:** 1 Complexity and Resilience Research Group, SBA Research gGmbH, Floragasse 7/5.OG, 1040 Vienna, Austria; sraubitzek2@sba-research.org (S.R.);; 2 Institute of Information Systems Engineering, TU Wien, Favoritenstrasse 9-11/194, 1040 Vienna, Austria; 3 Research Group Security and Privacy, University of Vienna, Kolingasse 14-16 5.OG, 1090 Vienna, Austria; 4 Josef Ressel Zentrum für Blockchain-Technologien und Sicherheitsmanagement, FH St. Pölten, Arbeitsplatz B—Campus Platz, 3100 St. Pölten, Austria; 5 Data Science Group, TU Wien, Favoritenstrasse 9-11/194, 1040 Vienna, Austria

**Keywords:** assembly theory, autocatalytic sets, complexity, formation of molecules, origin of life, autocatalysis

## Abstract

Assembly Theory provides a promising framework to explain the complexity of systems such as molecular structures and the origins of life, with broad applicability across various disciplines. In this study, we explore and consolidate different aspects of Assembly Theory by introducing a simplified Toy Model to simulate the autocatalytic formation of complex structures. This model abstracts the molecular formation process, focusing on the probabilistic control of catalysis rather than the intricate interactions found in organic chemistry. We establish a connection between probabilistic catalysis events and key principles of Assembly Theory, particularly the probability of a possible construction path in the formation of a complex object, and examine how the assembly of complex objects is impacted by the presence of autocatalysis. Our findings suggest that this presence of autocatalysis tends to favor longer consecutive construction sequences in environments with a low probability of catalysis, while this bias diminishes in environments with higher catalysis probabilities, highlighting the significant influence of environmental factors on the assembly of complex structures.

## 1. Introduction

The search for a comprehensive framework to discuss and describe complexity remains ongoing, despite the development of numerous theories over the years. Complexity science, which seeks to understand the behaviors and properties of complex systems, has introduced various concepts such as emergence, self-organization, and non-linearity. These have been instrumental in fields ranging from biology to economics. However, contemporary challenges—such as modeling global climate change, understanding large-scale social dynamics, and managing highly interconnected technological systems—continue to test the limits of existing frameworks [1,2]. This ongoing evolution of ideas underscores that our quest to understand complexity is far from complete.

Among the myriad theories that serve to describe the formation of complex structures are Assembly Theory and the theory of autocatalysis, which are the focus of this work. We must disclaim at this point that although the following discussion is framed within the context of chemistry, as was the original context of these frameworks, this article addresses these concepts on a more abstract level.

Assembly Theory provides a quantitative measure of molecular complexity through the molecular assembly (MA) index, which can be experimentally verified using mass spectrometry. This framework is crucial for identifying complex molecules that signify life, both on Earth and potentially on other planets. It posits that living systems produce complex molecules in abundance, which are highly unlikely to be created randomly or abiotically [3]. This MA index differentiates between biological and non-biological molecules, offering a robust, experimentally tractable measure of complexity that does not rely on specific biochemical pathways [3,4].

On the other hand, autocatalytic sets and autocatalysis in general are fundamental in understanding biochemical networks and the origin of life. These sets consist of chemical reactions where the catalysts for the reactions are produced by the reactions within the set itself, creating a self-sustaining system. Introduced by Stuart Kauffman in the early 1970s, autocatalytic sets have been pivotal in exploring the emergence and evolution of complex biochemical systems [5]. Further developments by researchers like Eigen and Schuster have emphasized the robustness and evolutionary potential of these networks [6].

The motivation for integrating Assembly Theory and the concept of autocatalysis lies in their complementary approaches to understanding complexity. Assembly Theory discusses pathway probabilities, while autocatalytic sets highlight the necessity for increased pathway probabilities to sustain certain biochemical processes. By merging these concepts, we aim to explore whether the principles of autocatalysis influence the fundamental pathways proposed by Assembly Theory. This integration is essential for a deeper understanding of how complex structures form and evolve.

**Figure 1:**
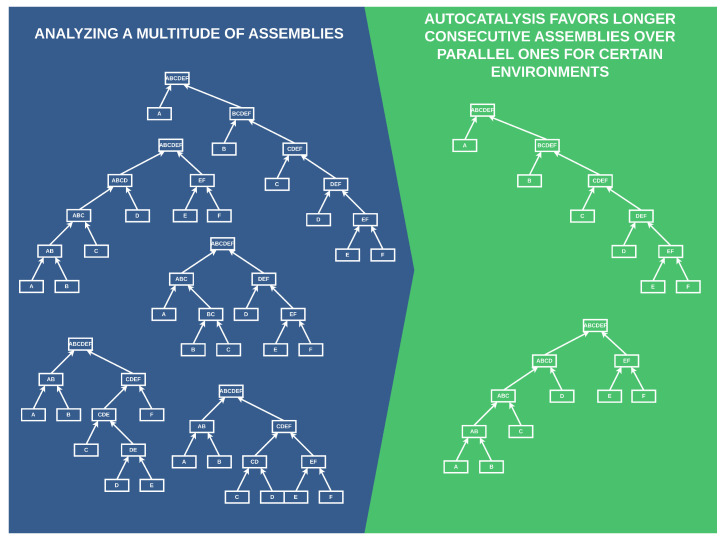
A simplified preliminary depiction of our results. Autocatalysis favors more consecutive builds of complex objects in environments with low catalysis. The left side portrays possible ways to form a complex object “ABCDEF”, and the right side shows the builds favored by autocatalysis.

This work aims to merge these two concepts and discuss their interplay using a simplified Toy Model. We demonstrate that autocatalysis impacts even the basic foundations of the connections that constitute complex structures. Our results illustrate that the likelihood of autocatalytic processes affects the pathways through which complex structures are formed. We achieve this by constructing a simplified Toy Model, analyzing its fundamental properties, and providing arguments on how and where our findings can be applied to real-life complexity. Our approach and the most significant results are presented in Figure 1.

Specifically, we analyze multiple ways to form a complex object and find that in environments where the probability of catalyzing necessary processes is low, autocatalysis favors more consecutive rather than parallel builds of complex objects.

This article is structured as follows. Section 2 provides a brief review of historical sources on autocatalytic sets and Assembly Theory in chronological order for readers to obtain an overview of key developments. Section 3 describes the employed concepts and tools from both Assembly Theory and autocatalytic sets. Section 4 introduces the developed Toy Model and discusses the assumptions and technical details involved. Next, we discuss our numerical experiments and results in Section 5, followed by an in-depth discussion in Section 6 and a final conclusion in Section 7.

## 2. Related Work

This section is a collection of past research that we consider as key developments and/or neat resources for the interested reader to obtain an overview of both Assembly Theory and the implications of autocatalysis.

### 2.1. Assembly Theory

Assembly Theory, as a framework for understanding the formation and complexity of systems, has received significant attention across various scientific disciplines, from biological to chemical and even abstract mathematical contexts.

But what is it actually about? Assembly Theory, conceived by Sara Walker, Lee Cronin, and their team, has been around for several years (Marshall et al. [3,7,8]; Liu et al. [9]) and has already sparked controversy in disciplines such as complexity science [10].

Originally developed as a tool for detecting biosignatures in the atmospheres of distant exoplanets [3,8], Assembly Theory offers a simplified, rule-based framework for understanding the combinatorial processes that drive innovation in abstract worlds of recombining elements. It serves as an example of computational complexity theory, particularly in its application to the emergence of complex structures. By applying its metrics to natural systems, Assembly Theory enables researchers to determine whether higher levels of organization, beyond the fundamental laws of physics, have emerged.

However, these are bold claims, and the work by Jaeger provides a more grounded perspective, offering clear arguments to temper the expectations surrounding Assembly Theory [11]. In essence, Jaeger boils it down to the following: *What Assembly Theory really does is detect and quantify bias caused by higher-level constraints in well-defined rule-based worlds.*

This is precisely how we apply Assembly Theory in this work—to identify biases resulting from autocatalysis in sampled assembly spaces.

Below is a compilation of key works for readers interested in Assembly Theory and the concept of autocatalysis.

The foundational concept of Assembly Theory is detailed in [12], where the authors introduce the notion of pathway assembly. This graph-based approach quantifies the number of steps required to assemble an object based on its hypothetical formation history, estimating the likelihood of its formation without biological processes. This early work provides a novel measure to distinguish randomly occurring objects from biologically assembled ones, setting the stage for later developments in Assembly Theory.

Building upon these early theoretical constructs, the authors of [4] discuss how Assembly Theory quantifies selection and evolution, particularly in biological systems. This work provides the theoretical underpinning for understanding how complexity can arise and be quantified in various contexts, establishing a foundation for subsequent research. Further, Reference [7] introduce a probabilistic framework for identifying biosignatures using pathway complexity. This approach allows for a more nuanced understanding of complex structure formation and identification, adding depth to deterministic views.

Theoretical advancements have significantly expanded the framework of Assembly Theory. The authors of [3] provide a comprehensive mathematical framework, focusing on constructing objects from basic elements within defined assembly spaces. This innovative method differentiates between objects formed by random processes and those resulting from directed biological or technological actions, offering a novel metric for assessing complexity and origins. Complementing this, Reference [3] introduce the concept of assembly spaces, providing a formalized approach to understanding the pathways leading to life’s formation, emphasizing the predictive power of Assembly Theory. Reference [9] explores the chemical space with molecular assembly trees, providing tools to map and navigate the vast possibilities within chemical spaces. This paper is crucial for extending the utility of Assembly Theory beyond biological contexts, demonstrating its relevance in chemistry and material science. Finally, Reference [13] delve into the assembly pathways of binary messages, showcasing the versatility of Assembly Theory in abstract mathematical contexts. This study provides a mathematical basis for understanding information assembly and hints at potential applications in computational sciences and information theory.

On the experimental front, several studies have applied Assembly Theory to practical scenarios. The authors of [8] introduce a novel approach for detecting extraterrestrial life by using mass spectrometry to measure molecular complexity through the molecular assembly index (MA). They validate this method on several million molecules, aiming to establish a universal criterion for identifying biosignatures, which is essential for both space missions and synthetic biology experiments on Earth. The authors of [14] present an experimental framework for quantifying molecular complexity, making significant strides in the direct measurement of MA using spectroscopic techniques. This approach not only enhances our understanding of molecular evolution but also holds potential for broad applications in drug development and origin-of-life research. The authors of [8] apply Assembly Theory to mass spectrometry, providing a practical framework for identifying molecules as biosignatures, thus bridging theoretical constructs and practical applications. Their study showcases how Assembly Theory can identify complex molecular structures that might indicate the presence of life. Additionally, Reference [4] provides empirical evidence and quantitative metrics for selection and evolution across biological and physical systems. They introduce a refined method for calculating the assembly index, demonstrating its practical application in distinguishing between random and directed processes. This work underscores the theory’s utility in experimentally measurable contexts, highlighting its relevance for both theoretical exploration and practical applications in understanding complex system formation.

Notably, the theoretical advancements, such as those presented by [3], complement the experimental insights provided by [8]. These studies collectively emphasize the predictive power of Assembly Theory and its structured approach to exploring the theoretical spaces within which complex structures can form. Together, these papers form a comprehensive overview of Assembly Theory’s applicability across multiple domains. They collectively highlight the theory’s potential to explain and predict complex structures in both natural and artificial systems, making a significant contribution to our understanding of complexity and the processes leading to the formation of life and other complex entities.

### 2.2. Autocatalysis

Autocatalysis, a concept central to the origin of life, has been extensively studied and developed over the years. Autocatalysis plays a fundamental role in the origin of life and chemical evolution. Despite its importance, a unified framework for studying autocatalysis has been lacking. Initially, the idea was independently introduced several times in the context of early life models [5,15,16]. The foundational work of Manfred Eigen and Peter Schuster on the hypercycle in the late 1970s introduced the idea of self-replication in molecules, a process crucial for biological systems. Although primarily biological, the hypercycle concept provides insights into general principles of complex systems that extend to other domains [16,17]. Building on this, Stuart Kauffman explored the formation of autocatalytic sets. He posited that life emerges as a phase transition, suggesting that given a sufficiently diverse mix of molecules, the formation of an autocatalytic system, which is self-maintaining and self-reproducing, becomes almost inevitable [18]. The core principle involves chemical reaction systems (CRSs), where a set of molecule types and reactions form self-sustaining networks that can grow and evolve. This is formally captured in the Reflexively Autocatalytic and Food-generated (RAF) theory, which defines an autocatalytic set as a subset of reactions where every reaction is catalyzed by molecules produced within the set, and all reactants can be generated from a basic food set through these reactions [19,20].

A significant milestone in the development of RAF theory was the introduction of the binary polymer model by Kauffman [5], where molecule types are represented as bit strings, and reactions involve the ligation and cleavage of these strings. This model helped demonstrate that autocatalytic sets can emerge with high probability under realistic levels of catalysis [5,21]. Subsequent work by Hordijk and Steel further refined the mathematical framework, proving that a linear growth rate in catalysis suffices to form RAF sets, and developing efficient algorithms to detect such sets within CRSs [21,22]. Empirical studies have shown that RAF sets are not necessarily large; they often consist of smaller, interdependent subsets called subRAFs, which can themselves be autocatalytic. This structural decomposability has profound implications for the evolvability and robustness of these systems. For example, an autocatalytic set can potentially be broken down into irreducible RAFs (irrRAFs), each capable of sustaining autocatalysis independently. This hierarchical organization allows for complex behaviors such as inheritance, mutation, and competition, akin to biological evolution [20,23]. The notion of RAF sets has expanded beyond origin-of-life studies to other fields like ecology and economics, where systems can be seen as networks of interdependent entities catalyzing each other’s production and survival [19,20]. For instance, in economics, production processes involving raw materials, intermediates, and catalysts (such as machinery) form autocatalytic-like networks, highlighting the universality and broad applicability of RAF theory [21]. Similarly, Lorenzo Napolitano’s analysis of technological networks reveals that innovation and patenting dynamics are shaped by autocatalytic structures. These structures grow as interconnected technological fields mutually benefit from their connections, reflecting a self-reinforcing pattern of innovation [24]. Also, Wim Hordijk’s work on the emergence of autocatalytic sets in models of technological evolution shows that these sets can form in simple models, providing insights into economic models and innovation dynamics [25]. Mike Steel’s concept of the adjacent possible complements these ideas by describing how human creativity involves combining existing items in new ways to generate further growth. This combinatorial approach is fundamental to innovation and can explain the accelerating pace of innovations. Steel’s model suggests that the adjacent possible refers to new opportunities that arise given the current state of existence [26].

The recent work by Kauffman and Roli further extends this by proposing that the emergence of life is an expected phase transition in the evolving universe. This theory unites the concepts of Collectively Autocatalytic Sets (CAS) and the Theory of the Adjacent Possible (TAP). In this context, TAP theory suggests that as the diversity and complexity of molecules increase, a phase transition occurs, leading to the spontaneous formation of molecular reproduction systems [27]. This integration provides a comprehensive view of how life can emerge from non-living matter through a predictable and inevitable process driven by increasing molecular complexity and catalytic interactions. The concept of catalytic closure, where every reaction in the system is catalyzed by at least one molecule within the system, is central to this theory and aligns with the principles outlined in RAF theory. This work not only reinforces the importance of autocatalysis in the origin of life but also suggests new avenues for research in astrobiology and the search for life on exoplanets by identifying potential catalytic networks [23,27].

Overall, the development of autocatalytic sets and RAF theory represents a significant leap in understanding the emergence and evolvability of complex systems, offering insights into the fundamental processes that underpin life’s origin and its diverse manifestations.

We also want to highlight the work by Blokhuis et al. [28], which provides a systematic classification of minimal autocatalytic motifs, referred to as *autocatalytic cores*. These motifs are the basic building blocks of all known autocatalytic systems, and new motifs are identified through this classification. The study also extends the range of autocatalytic systems by considering the emergence of autocatalysis from coupled compartments.

Autocatalysis is defined as a process where a substance catalyzes its own formation, which is crucial for self-replication in living systems. The authors propose general stoichiometric conditions to determine whether a subnetwork within a larger chemical network can exhibit catalytic or autocatalytic behavior. Autocatalytic networks are classified into five categories based on the nature of their reaction motifs. These classifications allow for the identification of novel autocatalytic cycles, some of which were previously unknown.

## 3. Methodology

Assembly Theory provides a framework for quantifying the complexity of molecular structures by measuring the information required to assemble them. On the other hand, the concept of autocatalysis often arises when dealing with chemical reactions in organic matter, leading to the formation of complex structures. This section introduces the mathematical tools from Assembly Theory, focusing on the Assembly Index and pathway probability. Additionally, we briefly introduce the concept of autocatalysis.

### 3.1. Assembly Index (AI)

The Assembly Index is a measure of the complexity of a molecular structure, defined as the minimum number of steps required to construct the molecule’s graph by recursively combining simpler substructures. To illustrate this, consider the example of constructing the word “ABRACADABRA” from its constituent letters.

- The word “ABRACADABRA” consists of 11 characters but only 5 unique letters: {A,B,R,C,D}.
- Begin with the letter “*A*”. Combine “*A*” with “*B*” to form “AB”. This is the first step. That is, we allow only for the following operation where two objects, not necessarily unique letters, are joined: [X]+[Y]→[XY], where [X] and [Y] can be “*A*”, “AB”, “ABRA”, “ABBA”, “ACAB”, or any other combination of unique letters of arbitrary length.
- Repeat this process to form “ABRA” in subsequent steps.
- The structure “ABRA” can then be reused to complete the word in fewer steps than starting from scratch each time.

We took this example from http://www.molecular-assembly.com/ (accessed on 18 September 2024) the Assembly Theory homepage [29]. The fewest number of steps, required to assemble “ABRACADABRA” by combining two components is thus 7, as detailed below:

(1) $$\begin{array}{c} \mathrm{Step}\;1:\;A+B\to AB\\ \mathrm{Step}\;2:\;AB+R\to ABR\\ \mathrm{Step}\;3:\;ABR+A\to ABRA\\ \mathrm{Step}\;4:\;ABRA+C\to ABRAC\\ \mathrm{Step}\;5:\;ABRAC+A\to ABRACA\\ \mathrm{Step}\;6:\;ABRACA+D\to ABRACAD\\ \mathrm{Step}\;7:\;ABRACAD+ABRA\to ABRACADABRA \end{array}$$

The assembly process involves constructing an object by sequentially joining a finite set of basic components. In this process, once an object is constructed, it can be reused in subsequent steps to form more complex structures. This process occurs within a defined framework known as the assembly space. The assembly space provides a formal structure in which all possible assembly pathways can be explored. It defines the relationships between basic components and the more complex structures they can form. The assembly space is essentially a map of all potential ways an object can be assembled, starting from its simplest components and leading to the final complex structure.

### 3.2. Linking Assembly Space, Molecular Assembly Index, and Pathway Probability

The concept of an assembly space is directly connected to the molecular assembly index and pathway probability. The molecular assembly index quantifies the complexity of a structure by determining the minimum number of steps needed to assemble it from basic components within the assembly space. This index reflects how “simple” or “complex” it is to build the structure, with a higher index indicating a more complex assembly process. Pathway probability comes into play when considering the likelihood of a specific assembly pathway being followed in the assembly space. Not all pathways are equally likely; some are favored due to factors like autocatalysis, as discussed in this article, which can bias the assembly process toward certain sequences of steps. A higher pathway probability suggests that a particular sequence of assembly steps is more likely, potentially indicating a non-random or biological origin for the structure [3]. In summary, the assembly space provides the foundational framework for understanding the construction of complex objects. The molecular assembly index quantifies the complexity of this construction process, while pathway probability evaluates the likelihood of different assembly pathways within the space, helping to distinguish between abiotic and biotic origins of complex molecules [3].

### 3.3. Autocatalytic Sets

The concept of autocatalysis is frequently discussed in the expanding body of literature modeling the emergence of self-sustaining biochemical systems essential for life [20]. It directly ties into the origin of life and the possibility of life forming by chance, given specific environmental conditions that catalyze chemical reactions, leading to the production of increasingly complex molecules and interactions. A formal definition of autocatalysis is often given in terms of RAF sets. Given a catalytic reaction system (CRS)—a network of molecule types and catalyzed chemical reactions—a (sub)set *R* of such reactions (plus the molecules involved in the reactions in *R*) is called

1. Reflexively autocatalytic (RA) if every reaction in *R* is catalyzed by at least one molecule involved in any of the reactions in *R*;
2. F-generated (F) if every reactant in *R* can be constructed from a small “food set” *F* by successive applications of reactions from *R*;
3. Reflexively autocatalytic and F-generated (RAF) if it is both RA and F.

**Figure 2:**
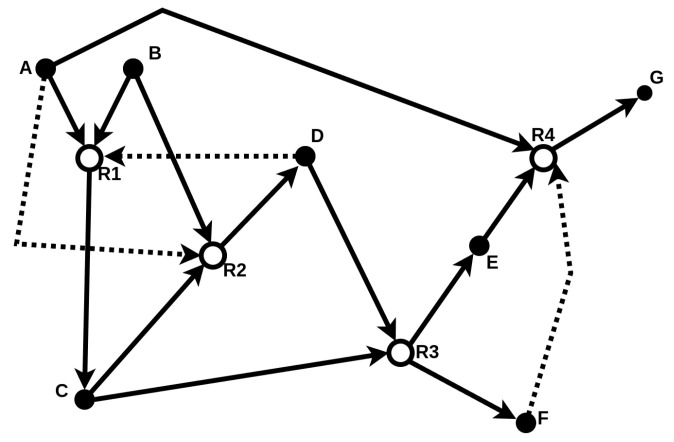
Schematic illustration of a catalytic reaction system (CRS) consisting of molecule types {A,B,C,D,E,F,G} (solid nodes) and four reactions {R1,R2,R3,R4} (open nodes). The food set is $\hat{F}=\left\{A,B\right\}$. Solid arrows represent reactants entering and products leaving a reaction, while dashed arrows represent catalysis. The subset $\hat{R}=\left\{R1,R2\right\}$ (highlighted with bold arrows) forms an RAF set. This example is taken and adapted from [20].

The food set *F* contains molecules assumed to be freely available in the environment. Therefore, an RAF set formally captures the notion of “catalytic closure”, i.e., a self-sustaining set supported by a steady supply of (simple) molecules from some food set [20]. A simple example of such an RAF set is provided in Figure 2.

Summed up, in this context, autocatalysis refers to a process where a chemical reaction is catalyzed by one of its own products, enabling a self-sustaining chain or network of reactions. This concept is central to understanding the origin of life, as it suggests that under the right conditions, simple molecules can catalyze the formation of increasingly complex systems, leading to the emergence of life. Additionally, the framework presented by Blokhuis et al. [28], extends the diversity of known autocatalytic systems by identifying minimal motifs that form the building blocks of more complex networks. These motifs allow for the formation of increasingly intricate systems under the right conditions.

## 4. A Toy Model for Autocatalytic Sets

**Figure 3:**
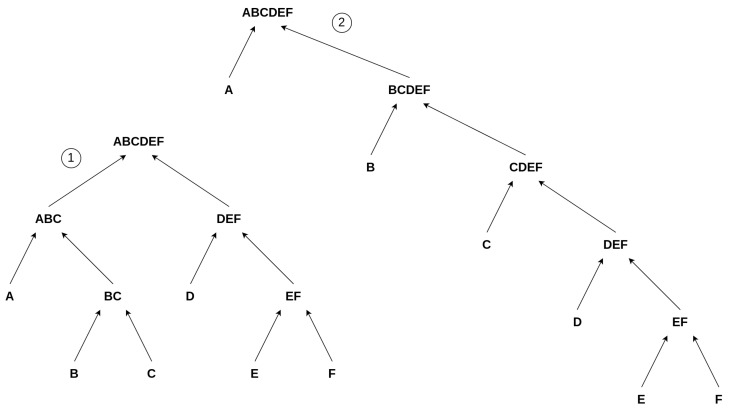
Suggested Toy Model: Two possible pathways/constructions to create the object “ABCDEF” within the confines of our model. The left construction, ①, depicts a more parallel construction, whereas the right one, ②, illustrates a more consecutive construction of “ABCDEF”.

We developed a Toy Model to discuss aspects of Assembly Theory and autocatalytic sets within one framework. Building upon the earlier example from Section 3.1, i.e., the “ABRACADABRA” construction, we simplify the model by avoiding repetitions. This means we build words of only unique letters. For example, we consider a word of length 6, “ABCDEF”, and explore its various constructions by always merging two constituents, as illustrated in Figure 3. However, this suggests hybrid model conflicts with previous research and corresponding foundations on Assembly Theory, as, in order to assemble a complex object, one is allowed to use already existing objects at each step of the construction [3,30,31,32]. For example, adding the already existing “ABRA” to the consecutive construction to form “ABRACADABRA” in step 7 in Equation (1). We avoid using already existing elements in the chain, allowing only unique constituents of an object. This simplification is necessary for our Toy Model to gain an initial understanding of the problem at the most fundamental level, in the most sterile scenario, and to determine if a connection between Assembly Theory and autocatalytic sets exists at this level. Our approach should be expanded in future work, for example, to include cases similar to Step 7 from Equation (1), allowing for greater complexity and variety among the covered symbolic reactions.

**Figure 4:**
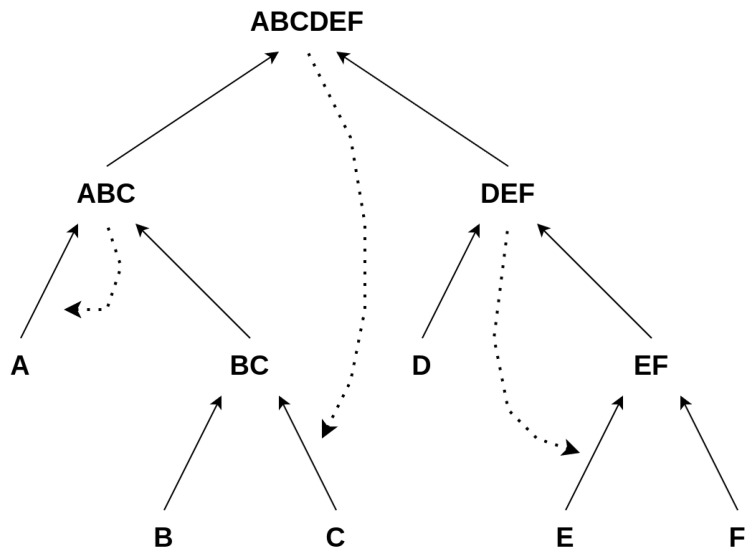
The depiction of how different reactions within the Toy Model can be catalyzed. For example, we can choose catalysts for a reaction from all products and constituents available in the entire chain/tree of reactions. The dashed lines show which reactions are catalyzed by which element, with the arrows pointing to the catalyzed reactions and the dashed lines starting at the chosen catalyst.

Next, we assign catalysts to our scheme/diagram (or suggestion) of reactions/events, as shown in Figure 4. This assignment is governed by probability, meaning we randomly determine whether a catalyst is assigned to a reaction with a probability from the set {0,0.1,0.2,…,0.9,1.0}. After determining if a reaction is catalyzed, we choose the catalyst from all elements involved in the reaction. We consider two methods for selecting the catalyst:

1. We choose an all-equally random catalyst. Further, we do not allow this catalyst to be used again.
2. We choose a catalyst with a weighted probability and allow catalysts to be used more than once.

**Figure 5:**
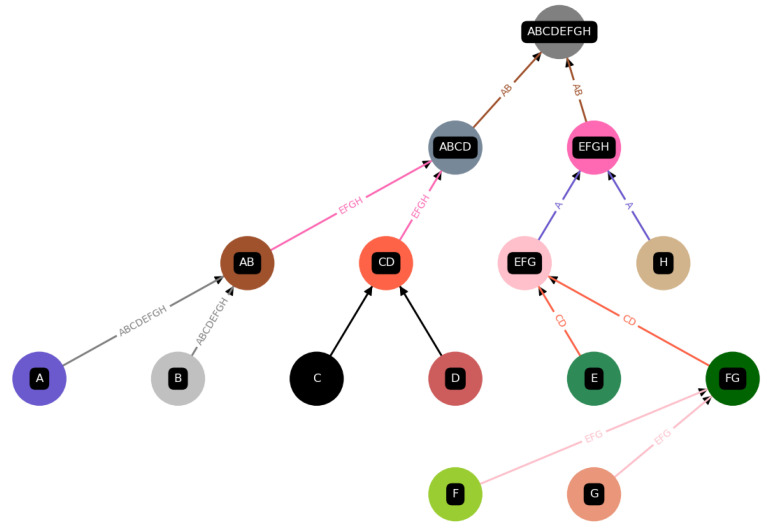
An illustration of one of the many possible ways to assemble “ABCDEFGH”, i.e., one construction among all possible ones in the assembly space for “ABCDEFGH”. This graph is a direct output from the code implementation (Python) of the Toy Model. Each product and component is assigned a unique color, and if that item is used as a catalyst, we also use that color for the arrows depicting the reaction.

The result of building a tree of reactions and randomly assigning a catalyst is shown in Figure 5, illustrating a direct output obtained by our code, which can be found in a Github repository (github.com/Autocatalytic-Sets-and-Assembly-Theory, accessed on 18 September 2024).

Summed up, we are creating random construction schemes/trees/*constructions* for an object/*final product*, e.g., “ABCDEF”, from its unique building blocks “A”, “B”, “C”, “D”, “E” and “F”, and all interactions are always sorted in alphabetical order to further simplify the model. All possible constructions are the assembly space of an object/final product. Thus, we formally describe the suggested model in the following way: For each final product *X*, which consists of $n_{X}$ unique elements/letters $\left\{\bar{x_{1}},\bar{x_{2}},\ldots ,\bar{x_{n_{X}}}\right\}$ and a corresponding construction $\Phi_{X}$, with $n_{X}-1$ reactions, and $n_{X}-2$ intermediate products $\left\{x_{1},x_{2},\ldots ,x_{n_{X}-2}\right\}$, we build up the final product *X* from the unique elements. Next, for each final product *X*, we can find the minimum depth, i.e., the corresponding depth for a maximal parallel distribution in the manner of ① from Figure 3, via the following relation, which is independent of the construction Φ, analogous to binary search trees, [33,34]:

(2) $$\mathrm{minDepth}\left(X\right)=\left\lceil \log_{2}n_{X}\right\rceil ,$$

where again $n_{X}$ is the number of unique elements of *X*. Thus, this minimal depth of each final product *X* is similar to the assembly index of a complex molecule as discussed by Assembly Theory [3,8]. In this construction Φ, we find a set of constituents and a set of products. The products

(3) $$P_{X}=\left\{X,x_{1},x_{2},\ldots ,x_{n_{X}-2}\right\},$$

contain the final product *X* and all elements created up to the final product $x_{j}$, but not the unique elements ${\bar{x}}_{i}$. In contrast, the constituents

(4) $$C\left(\Phi_{X}\right)=\left\{x_{1},x_{2},\ldots ,x_{n_{X}-2},{\bar{x}}_{1},{\bar{x}}_{2},\ldots ,{\bar{x}}_{n_{X}}\right\},$$

contain everything but the final product. Together, they form the set of all elements:

(5) $$E\left(\Phi_{X}\right)=P\cup C=\left\{X,x_{1},x_{2},\ldots ,x_{n_{X}-2},{\bar{x}}_{1},{\bar{x}}_{2},\ldots ,{\bar{x}}_{n_{X}}\right\},$$

containing a total of $2n_{X}-1$ elements. All catalysts are then chosen randomly from this set of all elements and form the set of catalysts *K*, with a maximum possible number of catalysts equal to the number of reactions $n_{X}-1$. We use two ways to choose the catalysts randomly: first, equally random without using one catalyst twice, and second, allowing it to use catalysts more than once but with a weighted probability such that longer catalysts are more probable to be chosen, such that

(6) $$\begin{array}{cc} W\left(e\right)=\frac{\mathrm{len}(e)}{\max\left(\mathrm{len}\left[E\left(\Phi_{X}\right)\right]\right)}\; & \mathrm{individual}\mathrm{weights}\mathrm{for}\mathrm{each}\mathrm{element}e\in E\left(\Phi_{X}\right),\\ W\left[E\left(\Phi_{X}\right)\right]=\sum_{e\in E\left(\Phi_{X}\right)}W\left(e\right)\; & \mathrm{accumulated}\mathrm{weight}\mathrm{for}\mathrm{the}\mathrm{set}\mathrm{of}\mathrm{all}\mathrm{elements}E\left(\Phi_{X}\right),\\ P\left(e\right)=\frac{W(e)}{W\left[E\left(\Phi_{X}\right)\right]}\;\;\;\; & \mathrm{normalized}\mathrm{probability}\mathrm{of}\mathrm{choosing}\mathrm{an}\mathrm{element}e\mathrm{as}a\mathrm{catalyst}, \end{array}$$

where len(e) is the length of an element *e*, or a set of lengths for all elements $\mathrm{len}\left[E\left(\Phi_{X}\right)\right]$.

We define a construction for a final product to be autocatalytic if all catalysts are within the set of all elements, $K_{X}\subset E\left(\Phi_{X}\right)$, which is always the case if at least one catalyst is assigned. A possible construction for a given final product “ABCDEFGH” is depicted in Figure 5.

However, within each construction Φ, we find a construction $\phi_{j}$ for each intermediate product $x_{j}$ and corresponding sets $P_{j},C_{j},E_{j}$, and $K_{j}$, which contain all products, constituents, elements in general, and catalysts, respectively, for each intermediate product. For example, in the case of the intermediate product “EFG” from Figure 5, this would correspond to

(7) $$\begin{array}{c} P_{j}=\left\{\mathrm{EFG},\mathrm{FG}\right\}\\ C_{j}=\left\{E,F,G,\mathrm{FG}\right\}\\ E_{j}=\left\{\mathrm{EFG},\mathrm{FG},E,F,G\right\}\\ K_{j}=\left\{\mathrm{CD},\mathrm{EFG}\right\} \end{array}$$

**Figure 6:**
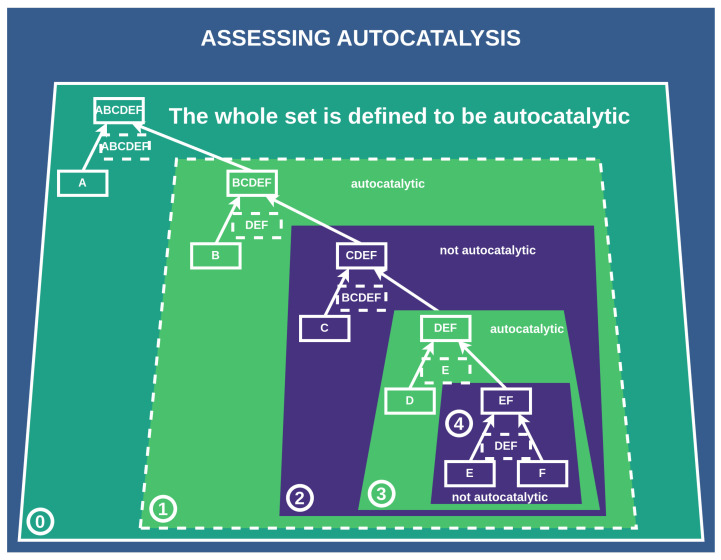
Assessing the autocatalysis of subnetworks: This scheme depicts how we assess whether a subnetwork/subconstruction of a construction is autocatalytic. Subconstructions with a purple shadow are not autocatalytic, as their catalysts are not contained within them. Conversely, subconstructions with a green shadow are autocatalytic, with all catalysts present among the products and constituents of the subconstruction. ⓪: The whole set is defined to be autocatalytic if at least one catalyst is assigned, which is true in this case. ①: We find this first subset to be autocatalytic since all catalysts are contained within it. ②: This subset is not autocatalytic since one of the catalysts BCDEF is not within the set of all elements of this subset. ③: Autocatalytic subset. ④: This subset is not autocatalytic since the catalyst DEF is not within this subset.

Here, the same definition for autocatalysis holds for every subconstruction $\phi_{j}$; i.e., all catalysts $K_{j}$ must be contained within the set of all elements $E_{j}$ for a subconstruction to be autocatalytic, which is obviously not the case for the above example, since the catalyst CD is not among the elements $E_{j}$, as illustrated in Figure 6

### Assembly Theory and Autocatalysis within the Toy Model

Next, we need to discuss the proposed model in an *Assembly Theory* context. This means we want to assess the assembly index, or the assembly properties of the regarded objects, and consider the pathway probability of each possible construction. However, the assembly index of each possible construction of a set of letters, e.g., “ABCDEF”, is trivial since the number of interactions/reactions/events necessary to construct this assembly of letters is always its length minus one, i.e., the steps it takes to construct this object. Thus, the assembly index of different words is

(8) $$\begin{array}{cc} \mathrm{AI}\left(ABC\right) & =2\\ \mathrm{AI}\left(ABCD\right) & =3\\ \mathrm{AI}\left(ABCDE\right) & =4\\ \mathrm{AI}\left(ABCDEF\right) & =5\\ \vdots & \; \end{array}$$

Still, there are different ways to build the objects, which are not captured by this rather strictly defined assembly index or by counting the number of interactions it takes to build an object but rather (just in our Toy Model) by a proxy for it, i.e., the depth of the set of reactions building up the final product “X”. We then analyze the distribution of these depths for a given element and determine the minimum depth as an approximation of the assembly index, as described in Equation (2). We depict these differences in depth in Figure 3. The first object, denoted ①, depicts a balanced structure where two strands, “ABC” and “DEF”, are constructed in parallel, corresponding to the minimal depth, Equation (2). The other chain of events is more consecutive, denoted as ②. Both chains of reactions have the same number of reactions, but the second chain (②) has an increased depth compared to ①. Comparing these two chains of events to our initial example of how to build “ABRACADABRA” (Equation (1)), we see that ① can be interpreted as a chain of events where, as in the “ABRACADABRA” case, a larger object joins at a later point in the reaction chain; i.e., “ABC” and “DEF” join to build “ABCDEF’‘. In contrast, ② depicts a more consecutive chain of events where no two larger objects are joining. Thus, we use the depth to characterize a construction and, consequently, the minimal depth as a proxy for the assembly index. Here, ① has a depth of 3 and ② a depth of 5. Thus, we differentiate between the individual constructions $\Phi_{X}$ by assessing the depth of a chain of reactions. The proxied “assembly index”, i.e., the minimal depth of this object, is 3, as you cannot construct this object without using a minimal depth of 3.

We also want to account for the pathway probability of each possible construction, and we carry this out by assessing the autocatalytic properties of a given catalyzed construction of an object. This means that each catalyzed construction of a given final product is autocatalytic such that all catalysts need to be part of the set of products and constituents, if at least one catalyst is set. Constituents are all elements that produce another one in a merging process, and products are all elements that are constructed by merging two constituents. Given our previous example from Figure 4, the set of all products is {ABCDEF,ABC,BC,DEF,EF} and the set of all constituents is {A,B,C,D,E,F,BC,EF,ABC,DEF}. We then count, for each construction, the number of sub-autocatalytic sets, meaning that we look at each sub-reaction/event and check if all used catalysts are contained within this set of products and constituents. This process is depicted in Figure 6. We deconstruct a catalyzed construction of an object into sub-objects and analyze the subsequent construction for being autocatalytic. For each final product and a particular construction, we increase the pathway probability of a particular construction based on its autocatalytic subset count. This is based on the intuition that if a certain process is autocatalytic, it is more likely to happen, as the corresponding set of reactions inherently amplifies itself. We implemented this in a way that adds multiples of the observed depth of a construction Φ to our distribution of Depths $D_{X}$ for all possible constructions Φ. First, we obtain the depth of a construction for each $\Phi_{X}$, i.e., $d\left(\Phi_{X}\right)$. This forms our initial case for building a distribution and a corresponding baseline—we collect all depths of a set of $n_{\Phi }$ random constructions Φ of a final product *X*. Next, we alter this distribution to account for autocatalysis by including each depth of a construction Φ $n_{\mathrm{autocatalysis}}$ times, where $n_{\mathrm{autocatalysis}}$ is determined such that

(9) $$n_{\mathrm{autocatalysis}}=\left\{\begin{array}{cc} 1, & \mathrm{no}\;\mathrm{autocatalytic}\;\mathrm{subsets}\\ {\left(1+k\right)}^{2}, & k\;\mathrm{autocatalytic}\;\mathrm{subsets} \end{array}\right.$$

We refer to this as exponential autocatalytic amplification below.

In sum, we analyze four distributions of depths across all constructions Φ for each final product *X*:

1. Equally randomly chosen catalysts, no weighting, no reuse;
  No autocatalytic amplification.
  This serves as our baseline distribution.
2. Equally randomly chosen catalysts, no weighting, no reuse;
  Exponential autocatalytic amplification.
3. Weighted randomly chosen catalysts, reuse allowed;
  No autocatalytic amplification.
4. Weighted randomly chosen catalysts, reuse allowed;
  Exponential autocatalytic amplification.

## 5. Numerical Experiments

Based on the previous description of our Toy Model and the corresponding assumptions in Section 4, we built an experimental setup to observe the influence of different catalyst probabilities and the presence of autocatalysis on the depth of a reaction chain leading to the formation of a particular object, and thus the final product. Specifically, we analyzed the distribution of different constructions that form a particular object within a sample assembly space consisting of 10,000 random constructions of that object, confined to the boundaries of the previously discussed Toy Model. We further modified the assembly space to include several copies of the construction in the presence of autocatalysis, according to Equation (9).

We tested different probabilities of catalysis, i.e., {0.0,0.1,0.2,0.3,0.4,0.5,0.6,0.7,0.8,0.9,1.0}, to observe how they influence the formation of different objects with and without the autocatalytic amplification of a particular construction scheme. For our experiments, we selected objects within a range of unique elements, starting from six unique elements “ABCDEF” up to 20 unique elements “ABCDEFGHIJKLMNOPQRST”. We tested the formation of these objects under various conditions and observed the average depth within the collected distributions of the assembly spaces to assess whether there is a bias in the construction of these objects and how this bias changes under different conditions.

### 5.1. Toy Model Implementation

The following section outlines the implementation of the previously discussed Toy Model. The Python code for this implementation is available at https://github.com/Raubkatz/Autocatalytic-Sets-and-Assembly-Theory (accessed on 18 September 2024).

Starting with the final product *X*, we recursively divide it into two parts until we construct a full tree of reactions, as illustrated in Figure 5. In the second step, we randomly apply catalysts to each reaction, either uniformly randomly or using weighted randomness. The Algorithm 1 below formally describes this procedure. We repeated these processes 10,000 times for each parameter set, including different conditions such as autocatalytic amplification, varying catalyst assignments, and a range of catalyst probabilities. We then analyzed the maximal depths of each reaction tree and calculated the average depths to identify potential biases in the sampled assembly spaces under different conditions, such as catalyst probabilities and the presence or absence of autocatalytic amplification.
**Algorithm 1** Creaqting a Construction $\Phi_{X}$**Algorithm to construct the autocatalytic sets from the proposed Toy Model Initialization:**• final_product ←X*Target product to synthesize, e.g., “ABCDEF”*• catalyst_probability $\leftarrow p_{\mathrm{cat}}$*Probability of assigning a catalyst*• switch_cat← strategy defined in Section 4, Equation (6)*Catalyst assignment strategy*• reaction_set ←∅*Set to store all reactions*• all_elements ←∅*Set to store all unique elements*• layer← 0**Recursive Product Decomposition:**  1: **Function:** split_product(product←final_product, layer)  2: Add product to all_elements  3: **if** length(product) = 1 **then**  4:  **return** *Base case: product is a single element*  5: **end if**  6: andomly split product into two reactants, $r_{1}$ and $r_{2}$  7: Add the reaction $\left\{\mathrm{product},r_{1},r_{2},\mathrm{layer}\right\}$ to reaction_set  8: split_product($r_{1}$, layer + 1)*Recursively process the first reactant*  9: split_product($r_{2}$, layer + 1)*Recursively process the second reactant***Catalyst Assignment:**  1: **for** each reaction $R_{i}$ in reaction_set **do**  2: Generate a random number *r* in the range [0.0,1.0]  3:  **if** r≤catalyst_probability **then**  4:   Choose $c_{i}\in \mathrm{all}\_\mathrm{elements}$      based on random or weighted random strategy set by switch_cat  5:   Assign catalyst $c_{i}$ from all_elements to $R_{i}$:      $R_{i}\leftarrow R_{i}\cup \left\{c_{i}\right\}$  6:  **end if**  7: **end for**  8: **return** reaction_set*The set of all reactions characterizes a construction $\Phi_{X}$*

### 5.2. Results

We obtained results for a range of different catalysis probabilities $p_{cat}\in${0.0, 0.1, 0.2, 0.3, 0.4, 0.5, 0.6, 0.7, 0.8, 0.9, 1.0} and a range of different object lengths $l_{\mathrm{object}}$ consisting of unique letters, where $l_{\mathrm{object}}\in Z$ and 6≤l≤20.

We further tested these conditions with and without amplification for autocatalysis and with and without a weighted catalyst assignment. This means we obtained a sample distribution of the assembly space for each probability (11), each length *l* (15), with and without catalysis amplification (2), and with and without catalyst weighting (2). Thus, 11×15×2×2=660 distributions were analyzed for their average construction depth, and we searched for biases within these distributions, particularly focusing on whether autocatalytic amplification influences these distributions and favors certain constructions over others.

**Figure 7:**
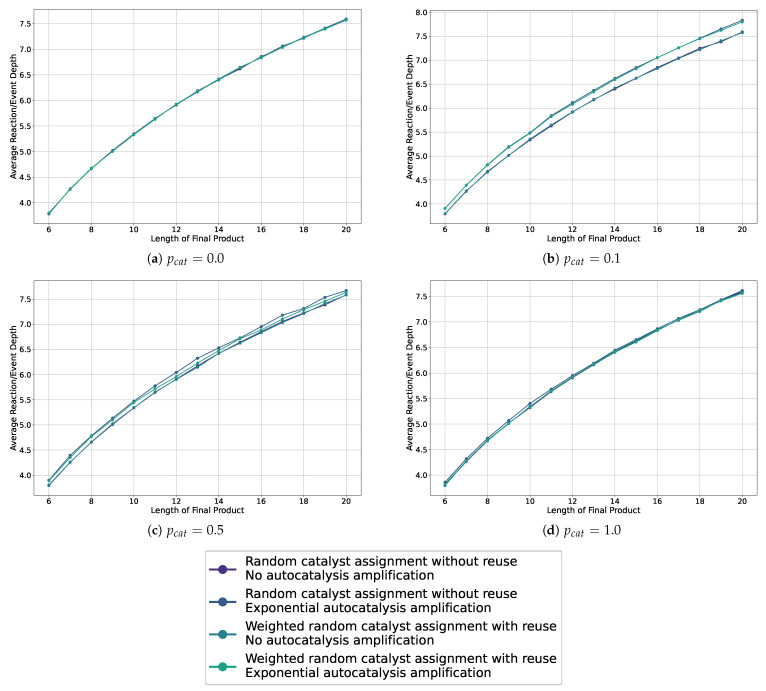
Line plots showing the average depth in the sampled assembly space for various settings, including weighted and non-weighted, and with or without autocatalytic amplification, across different probabilities of catalysis.

We show the average depths in the sample assembly spaces for probabilities 0.0, 0.1, 0.5, and 1.0, for all catalysis and autocatalysis configurations in Figure 7. Additional plots are collected in Appendix A. These graphics show that we do not observe a separation, and thus no bias, for autocatalysis or weighted catalysts when the catalysis probability is 0.0 or 1.0. Furthermore, across all plots, we do not see a difference or bias in how the catalysts are chosen—whether weighted or not. However, in the cases with probabilities 0.1 and 0.5, we observe a separation and thus a bias toward longer reaction depths for all cases with autocatalytic amplification, with the greatest separation observed for a catalysis probability of 0.1.

**Figure 8:**
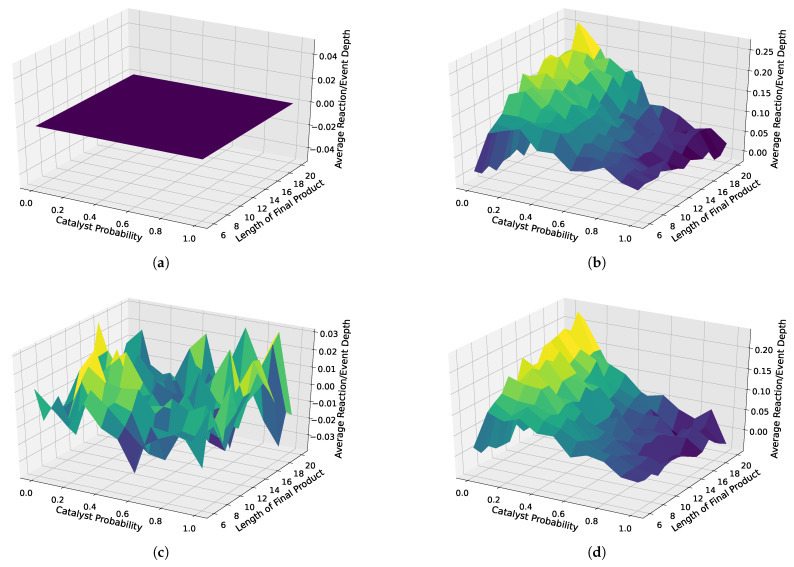
Three-dimensional surface plots for different scenarios: with and without autocatalytic amplification and with weighted and non-weighted catalysts. The upper left plot (**a**) is the baseline, which we subtracted from all other cases to highlight the differences, i.e., the separation from the baseline. The remaining plots show how different sampled assembly spaces vary in terms of the average reaction depth. (**b**) Random catalyst assignment without reuse, with autocatalytic amplification. (**c**) Weighted random catalyst assignment with reuse, without autocatalytic amplification. (**d**) Weighted random catalyst assignment with reuse, with autocatalytic amplification.

We also depicted the results for all analyzed distributions in 3D surface plots. Here, we used the case without weighted catalysts and no autocatalytic amplification as a baseline, which we subtracted from all three other cases, i.e., weighted catalysis and no autocatalytic amplification, weighted catalysts and autocatalytic amplification, and non-weighted catalysts with autocatalytic amplification. These 3D surface plots are depicted in Figure 8. First, as a proof of concept, we see that the baseline is zero, clearly indicating which case is used as a baseline. Next, we observe a strong separation, with the strongest separation occurring for a probability of 0.1, and increasing the separation for increasing object lengths, in both cases with and cases without weighted catalysts and autocatalytic amplification. However, the case with non-weighted catalysts shows slightly more separation. Furthermore, we do not observe any interpretable trends for the case without autocatalytic amplification.

Thus, given the previously discussed results presented in Figure 7 and Figure 8, we conclude the following.

- The autocatalytic amplification, which alters the distribution in the sampled assembly space for certain constructions/pathways, causes a bias toward longer chains of reactions/events and thus greater depths of the observed constructions.
- We cannot conclude that the weighting of the catalysts has any impact on the distribution of pathways in the observed sample assembly spaces.
- We observe an increasing separation of the average depths between cases with and without autocatalytic amplification in our sampled assembly spaces for lower probabilities of catalysis (i.e., highest for $p_{\mathrm{cat}}=0.1$) and increasing object lengths $l_{\mathrm{object}}$.

## 6. Discussion

Our results showed that autocatalysis can favor longer consecutive reaction chains, indicated by a bias towards increased average depths, rather than reaction chains occurring in parallel. This is somewhat evident from our definition of autocatalysis used in our model (Section 4). Specifically, if all catalysts are part of all reactions within the model, we consider it autocatalytic. This implies that organizing events into a longer consecutive chain increases the probability of incorporating all autocatalysts, as the cascading sequence encompasses most events. However, this bias disappears at different probabilities of catalysis. When the probability of catalysis is increased, this bias diminishes, allowing more distributed and less consecutive chains to be equally prevalent among our possible reactions.

To explain further, in environments with a low probability of catalysis, the preferred way of building complex, catalyzed objects is through a consecutive chain of events (e.g., A+B→AB,AB+C→ABC, etc.). In contrast, environments with a higher probability of catalysis favor more parallel or interconnected catalyzed reaction chains.

In terms of Assembly Theory, this suggests that the pathway probability of forming complex objects is closely tied to their environment. Analyzing a complex object without considering its environment might give a distorted view of the probability of a particular pathway. Though this study does not directly deal with an interpretable assembly index, we conclude that the dependency of pathways on environmental conditions (i.e., the probability of catalysis) could mean that the shortest or longest assembly index for a particular object might never be realized in certain environments. Thus, sampled assembly spaces and the statistics thereof might suffer from a bias toward more consecutive, and thus longer, chains of reactions and events in environments with a lower probability of catalysis. On the other hand, we might only observe the shortest chains of reactions and events in environments with a high probability of catalysis, e.g., biological environments developed to produce complex molecules. We thus conclude that the statistics of sampled assembly spaces are indicative of a certain environment, as these sampled assembly spaces might suffer from a bias toward longer reaction chains in environments with a low catalysis probability. Given certain edge cases, this might obscure the actual assembly index of the construction of a complex object by only slightly increasing constructions. Furthermore, this might enable the detection of autocatalysis indirectly by analyzing the statistics of different sampled assembly spaces of the same process/construction in different environments. This, of course, applies to the original use-case of Assembly Theory, i.e., the analysis of molecular structures [3]. However, we also want to provide an example in terms of software development.

Given an environment of a small team of software developers within a small company with very limited infrastructure, this team will have to build everything step by step themselves and cannot draw on results from other teams within the company. Such a team might, for example, build an initial implementation, then build a server structure to test it on, and so on. This corresponds to an environment with a low probability of catalysis, i.e., the team cannot draw on infrastructure (catalysts) from other teams or products; they need to build the catalysts themselves, which only become available to earlier steps afterward. Thus, in such an environment, a team of developers needs to follow a more consecutive chain of events/reactions. In a bigger company where a lot of infrastructure and other resources are available, a team aiming to build the same product might draw on infrastructure, e.g., available servers, at every step of the way to the final product, i.e., draw on more catalysts. Though not all processes are autocatalyzed, they are catalyzed within the company environment, and thus an environment like this can run more parallel builds, as products from a variety of teams are always available to each team.

Overall, we believe the presented work is a reasonable contribution to the growing body of both Assembly Theory and autocatalysis. However, to connect our results, or more precisely our ideas, with a more realistic scenario, one might apply our ideas, e.g., of increased pathway probabilities to RAF and TAP models and similar use-cases [20,25] and observe the distribution of the length of assemblies or another proxy for this within the generated networks—i.e., analyze sampled assembly spaces of, e.g., RAF and TAP models and conclude, e.g., with pathway probabilities from these approaches.

In a more complex build, we need to find out which pathways in the assembly are connected to autocatalysis and thus analyze the statistics of the assembly space, taking into account pathway probabilities to make pathways associated with autocatalysis visible.

Further, we want to mention a very speculative guess that Assembly Theory, and particularly this statistical analysis of sampled assembly spaces with corresponding pathway probabilities and assembly indices, might help in showing phase transitions for the emergence of life in unions of TAP and RAF models, as proposed in a recent work by Stuart Kauffman [27].

## 7. Conclusions

Our research aims to find a connection between autocatalysis in a reaction network and Assembly Theory, a relatively recent theory developed to characterize the complexity of objects, particularly molecules. We discuss both key concepts, i.e., Assembly Theory and autocatalysis in a given reaction network, and, based on the basic principles of these frameworks, develop a Toy Model that explores how certain objects of different complexities form in the presence of autocatalysis.

The developed Toy Model takes into account varying probabilities of catalysis in the construction of objects of different complexities, exemplified by sequences such as “ABCDEF’‘. It examines how the presence of autocatalysis in the construction of these objects influences the construction process by affecting the probability of different constructions or reaction/event chains leading to a certain object. We further assess the differences in individual construction schemata by using the depth of a given construction. The minimal depth of these reaction chains serves as a proxy for the assembly index, a key concept of Assembly Theory. After creating and analyzing a multitude of different objects and their constructions, i.e., a sampled assembly space, we observe that the presence of autocatalysis in these constructions introduces a bias toward a more sequential construction of complex objects. This finding aligns with Jaeger’s work [11], which highlights that Assembly Theory is particularly well suited for detecting bias in well-defined, rule-based systems/worlds.

This bias is somewhat evident because a more sequential construction naturally aligns with the concept of autocatalysis, where the elements necessary for building a certain object, along with the object itself, have an increased probability of catalyzing their own formation. In a sequential construction, each step in the chain directly builds upon the previous one, making it easier for a subset to be autocatalytic as the sequence progresses. Conversely, in a parallel construction, multiple strands of reactions may proceed simultaneously, with each strand potentially catalyzing others rather than catalyzing within its own sequence. This can result in fewer autocatalytic subsets within each individual strand, as the focus shifts away from auto/self-catalysis toward mutual catalysis between parallel strands. However, this bias vanishes for increasing probabilities of catalysis, indicating that while sequential constructions of complex objects are favored by autocatalysis in environments with scarce catalysis, this is not the case in environments with higher catalysis probabilities. We ultimately derive from our results that while sequential constructions of complex objects are favorable for initiating formation in simple environments, in more complex environments with abundant catalysis, this bias is less prominent, allowing for a more parallel assembly of complex objects.

In conclusion, our study demonstrates that the presence of autocatalysis in reaction networks can favor longer consecutive reaction chains over parallel chains, particularly in low-catalysis environments. This finding is crucial for understanding how the complexity of objects can be influenced by the probability of catalysis in their environment. This suggests that the pathway probability of forming complex objects is closely tied to its environment, and analyzing a complex object without considering its environmental conditions might provide a distorted view of the pathway’s probability and ultimately obscure the associated assembly index. We propose that analyzing the statistics of sampled assembly spaces could add information to the representation of the pathways forming complex objects, emphasizing the significance of autocatalytic reactions/connections/events within the environment.

## Figures and Tables

**Figure A1:**
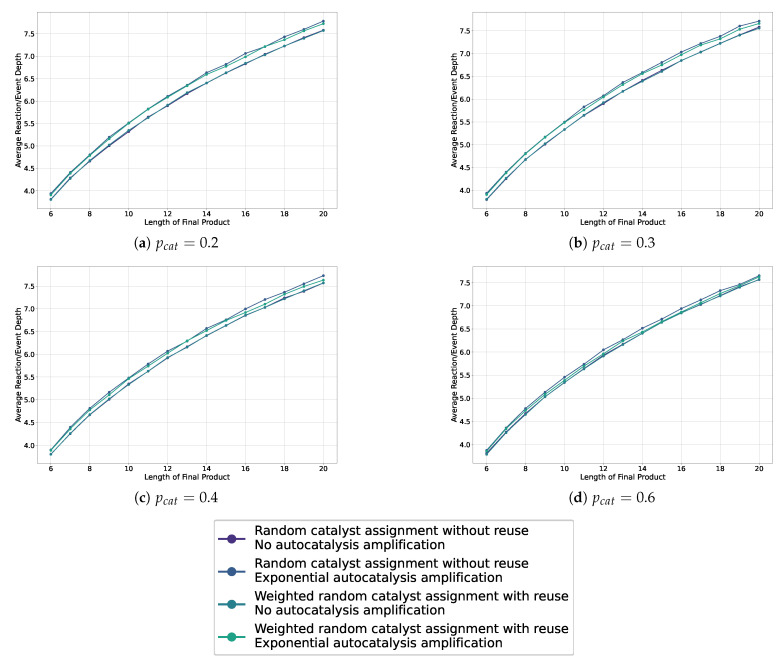
Line plots showing the average depth in the sampled assembly space for various settings, including weighted and non-weighted, and with or without autocatalytic amplification, across different probabilities of catalysis.

**Figure A2:**
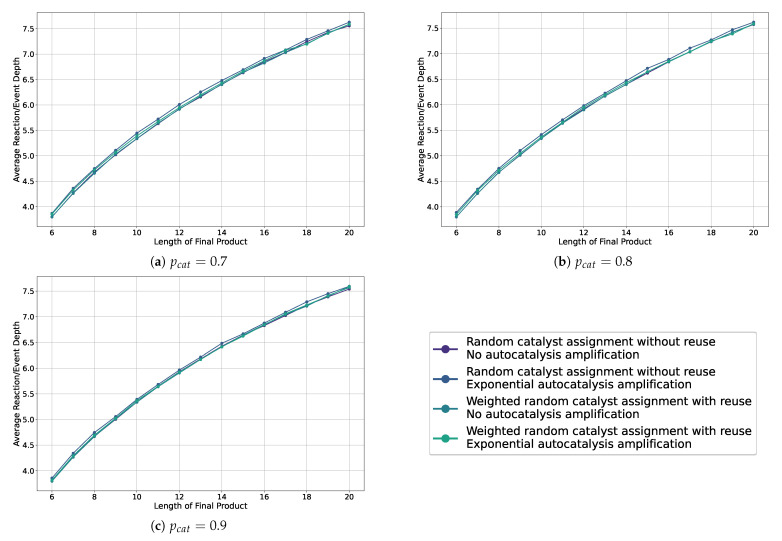
Line plots showing the average depth in the sampled assembly space for various settings, including weighted and non-weighted, and with or without autocatalytic amplification, across different probabilities of catalysis.

## Data Availability

The full program code to reproduce the presented experiments is available at https://github.com/Raubkatz/Autocatalytic-Sets-and-Assembly-Theory (accessed on 18 September 2024).

## Appendix A. Additional Plots

This Appendix collects the missing line plots from Section 5.2 for probabilities $p_{cat}\;\in \left\{0.2,0.3,0.5,0.6,0.7,0.8,0.9\right\}$.
